# ER Stress Induces Endothelial Dysfunction Linked to Mitochondrial Redox Imbalance and Enhanced Mitochondrial Bioenergetics in Penile Erectile Tissue

**DOI:** 10.3390/ijms27219761

**Published:** 2026-10-30

**Authors:** Alfonso Gómez del Val, Cristina Contreras, Lucía Olmos, Mercedes Muñoz, Óscar Freire-Agulleiro, Mariam Mohamed, Sara Benedito, Luis Rivera, Miguel López, Ana Sánchez, Dolores Prieto

**Affiliations:** 1 Departamento de Fisiología, Facultad de Farmacia, Universidad Complutense de Madrid, 28040 Madrid, Spain; 2 Departamento de Fisiología, CIMUS, Universidad de Santiago de Compostela, 15782 Santiago de Compostela, Spain

**Keywords:** ER stress, penile erectile tissue, mitochondrial ROS, endothelial dysfunction, mitochondrial respiration, Nox

## Abstract

Endoplasmic reticulum (ER) stress has been proposed as a link between insulin resistance, oxidative stress, and endothelial dysfunction in metabolic disorders. Penile erectile dysfunction shares risk factors with cardiovascular disease (CVD) including insulin resistance, diabetes, and obesity. In the present study, involvement of ER stress in penile endothelial and vascular dysfunction was investigated in an in vivo model of ER stress induced by tunicamycin. Endothelial function was assessed by using wire myography, mitochondrial reactive oxygen species (mtROS) by mitoSOX and Amplex Red fluorometry, mitochondrial bioenergetics by Seahorse XF Analyzer, and expression of ER-stress markers by Western blot. Tunicamycin increased expression of BiP, pPERK, ATF6α, and pIRE1α in penile erectile tissue. Endothelium-dependent relaxations to acetylcholine were impaired, while α_1_-adrenergic vasoconstriction was blunted in penile arteries from ER-stress rats. Selective NADPH oxidase 1 (Nox1) inhibition restored endothelial relaxations, while blockade of Nox2 and Nox4 revealed H_2_O_2_-mediated endothelial relaxations in control but not in tunicamycin-treated rats. ER stress reduced basal mtROS levels and mitoTEMPO- sensitive relaxant responses. On the other hand, enhanced mitochondrial respiration and glycolytic activity were found in penile arteries from tunicamycin-treated rats. These findings indicate that ER stress blunts adrenergic vasoconstriction, and induces endothelial dysfunction by enhancing Nox1-derived ROS and reducing mitochondrial vasodilator H_2_O_2_. Attenuation of ER stress emerges as a potential therapeutic target in the management of metabolic disease-associated erectile dysfunction.

## 1. Introduction

Endoplasmic reticulum (ER) is a cell organelle with multiple functions such as protein folding and assembly, lipid and sterol biosynthesis, intracellular Ca^2+^ storage, and cell signaling and organelle communication [1]. Disruption of ER protein folding causes accumulation of unfolded proteins in the ER lumen, which is referred to as ‘ER stress’, leading to the unfolded protein response (UPR) to maintain ER homeostasis [1]. Dysregulation of ER homeostasis is involved in the pathogenesis of metabolic disorders such as obesity and diabetes, in which UPR is activated because of excessive nutrient influx, concomitant with increased demand for protein synthesis required for metabolic processes and glucose deprivation due to insulin resistance [2,3]. Moreover, UPR can also be activated by pathological stimuli such as hypoxia, nutrient scarcity, aberrant glycosylation, calcium depletion, oxidative stress, DNA damage, and energy deficits [3,4].

Endothelial dysfunction is a key factor in the pathogenesis of the cardiovascular complications of metabolic disorders, mostly due to impaired nitric oxide (NO) synthesis and/or bioavailability elicited by oxidative stress, inflammation, and altered endothelial barrier function [5]. ER stress has been implicated in the development of endothelial dysfunction and accelerates the progression of atherosclerotic-lesion formation preceding the onset of diabetes-associated dyslipidemia, which suggests that ER stress is a contributing factor in the early pathophysiological events of diabetes [3,6]. Chemically-induced ER stress with tunicamycin in endothelial cells stimulates increased secretion of pro-inflammatory cytokines and chemokines involved in atherogenesis, such as monocyte chemoattractant protein 1 (MCP-1) [7], augments NADPH oxidase (Nox)-dependent oxidative stress, reduces NOS activity, and impairs vascular endothelium-dependent relaxations [8].

Penile erectile dysfunction (ED) shares risk factors with cardiovascular disease (CVD), such as age, insulin resistance, diabetes, obesity, hypertension, and dyslipidemia, and has therefore been considered a sentinel sign preceding CVD including coronary artery disease [9,10,11]. Penile erection is a hemodynamic process in which arterial dilation induced by NO derived from both nerves and endothelium is essential for the initiation and the sustained phases of erection, respectively. Therefore, blunted NO availability mainly due to oxidative stress in metabolic diseases plays a key pathogenic role in penile endothelial dysfunction [12,13,14]. Thus, reduced NO and altered NO-signaling pathways in both endothelium and nerves, exacerbated vasoconstriction, oxidative stress, and inflammation lead to the vascular complications associated with ED in diabetic patients and experimental models of diabetes and obesity [13,15,16].

Impaired interactions between ER and mitochondria have been implicated in endothelial dysfunction and oxidative stress underlying the cardiovascular complications of metabolic disorders [3,8,17]. We have recently demonstrated that obesity induced by high-fat diet (HFD) elicits endothelial dysfunction and alters mitochondrial bioenergetics in penile erectile tissue [18]. Information on the pathogenic role of ER stress in ED associated with metabolic disorders such as diabetes and obesity is limited. We hypothesize here that ER stress may be involved in endothelial dysfunction and impaired bioenergetics of penile arteries in metabolic disease, and therefore we sought to elucidate the specific contribution of Nox-derived ROS, changes in mitochondrial redox balance and mtROS levels, and impaired mitochondrial respiration to the ER stress-induced penile vascular dysfunction by using an in vivo rat model of ER stress chemically-induced with tunicamycin.

## 2. Results

### 2.1. ER Stress Induces Endothelial Dysfunction and Blunts ɑ_1_-Adrenergic Vasoconstriction in Penile Arteries

Chemical induction of ER stress in vivo in penile erectile tissue was determined by the expression of ER-stress markers in CC. Treatment with tunicamycin increased expression of BiP, pPERK, ATF6α, and pIRE1α in CC samples (Figure 1a–d). Metabolic parameters of ER-stress rats are provided in Table 1 showing no changes in either weight or triglycerides levels, but increased blood lactate and a trend to increase in blood glucose levels in tunicamycin-treated rats.

To evaluate the impact of ER stress on the contractile responses of penile arteries, concentration-response curves for Phe were performed, showing a significant reduction in both maximal contractile response and sensitivity to the α_1_-adrenergic agonist in tunicamycin-treated rats (pD_2_ values: 6.12 ± 0.30, *n* = 7 in controls, and 5.39 ± 0.21 in arteries from tunicamycin-treated animals, *n* = 11, *p* < 0.05) (Figure 2a). These data indicate impaired α_1_-adrenergic vasoconstriction in the ER-stress model, in contrast to contractions induced by depolarization with a high K^+^ solution (KPSS) that were not altered in arteries from ER-stress rats compared to controls (Figure 2b).

To determine the relationship between ER stress, oxidative stress, and penile endothelial dysfunction, endothelium-dependent vasodilation was first examined in arteries from tunicamycin-treated rats. Relaxations to the endothelial agonist ACh were reduced compared to controls, with pD_2_ values of 6.64 ± 0.16, *n* = 15 arteries from 11–12 control rats, and 5.61± 0.30, *n* = 15 arteries from 11–12 control ER-stress rats (*p* < 0.001, unpaired Student’s *t*-test) (Figure 2c,d). Relaxant responses elicited by the general β-adrenergic agonist isoprenaline, that are partially mediated by endothelial NO, were also reduced in arteries from the ER-stress rat model, pD_2_ values of 6.69 ± 0.26, *n* = 7 arteries from 6 control rats, and 6.16 ± 0.18, *n* = 9 from 6 tunicamycin-treated rats, (*p* < 0.01, unpaired Student’s *t*-test) (Figure 2e) These findings confirm endothelial dysfunction associated with ER stress in penile arteries.

### 2.2. Contribution of Nox-Derived ROS to Endothelial Dysfunction of Penile Arteries from ER-Stress Rats

The role of Nox-derived ROS was first assessed by treating the arteries with the non-selective Nox inhibitor apocynin (10 µM), which did not significantly improve endothelium-dependent relaxations in penile arteries from rats treated with tunicamycin (Figure 3a,b; Table 2). The selective Nox1 antagonist NoxA1ds enhanced maximal endothelial relaxations, and increased Emax without a statistically significant change in pD_2_ to ACh in ER-stress rats (Figure 3c,d, Table 2), while treatment with the selective Nox2 inhibitor Nox2ds-tat did not significantly alter blunted endothelial relaxations of tunicamycin-treated rats but revealed a Nox2-mediated endothelial relaxation in control arteries (Figure 3e,f, Table 2). These data indicate that penile endothelial dysfunction involves increased Nox1-derived superoxide, and the loss of Nox2-derived vasodilator ROS. Treatment with the Nox1/Nox4 inhibitor GKT136901 mimicked the restoration of endothelial relaxations elicited by Nox1 in ER-stress arteries and inhibited relaxations in control arteries (Figure 3g,h, Table 2).

### 2.3. Contribution of mtROS to Endothelial Dysfunction of Penile Arteries from ER-Stress Rats

Given the interactions between mitochondria and ER, the role of mitochondrial ROS in endothelial dysfunction of penile arteries from chemically induced ER-stress rats was further assessed. The mitochondrial antioxidant mitoTEMPO reduced relaxations to ACh (Figure 4a; Table 3) and isoprenaline (Figure 4c; Table 3) in control arteries, suggesting the involvement of mitochondrial vasodilatory H_2_O_2_. However, treatment with mitoTEMPO did not inhibit endothelium-dependent relaxations to either ACh (Figure 4b) or isoprenaline (Figure 4d), but rather induced an increasing trend in the sensitivity to ACh, although not statistically significant, in arteries from ER-stress rats.

Mitochondria-derived ROS levels, including superoxide and H_2_O_2_, were determined by using MitoSOX and Amplex Red fluorescence, respectively. Tunicamycin-treated rats showed significantly lower levels of mtROS in both CC and penile arteries compared to controls (Figure 4e,f), while basal H_2_O_2_ levels were also reduced in CC of the ER-stress model (Figure 4g). Expression of the transcription factor regulating mitochondria gene expression of antioxidant enzymes PGC1α (Figure 4i) exhibited a downwards trend in CC of ER-stress rats, while MnSOD protein levels were not significantly changed (Figure 4h).

### 2.4. ER Stress Enhances Mitochondrial Bioenergetics in Penile Arteries

To assess the impact of ER stress on the mitochondrial bioenergetics of erectile tissue, a mitochondrial stress test was performed in fresh intact penile arteries using Agilent Seahorse XF HS Analyzer. This procedure demonstrated a significant increase in basal respiration, oxygen consumption for ATP synthesis, proton leak (Figure 5a–d), and glycolytic activity (Figure 5e) in penile arteries from rats with chemically induced ER stress.

## 3. Discussion

ER stress seems to be the link between insulin resistance, oxidative stress, and vascular endothelial dysfunction in metabolic disorders [19,20]. In the present study, the involvement of ER stress in penile endothelial dysfunction and in the vascular alterations of metabolic disease-associated ED [12,18] was further clarified in an in vivo model of chemically-induced ER stress. Expression of ER-stress markers was significantly enhanced in erectile tissue of tunicamycin-treated rats, consistent with activation of UPR pathways, and associated with endothelial dysfunction, blunted adrenergic vasoconstriction, impaired mitochondrial redox balance, and enhanced mitochondrial bioenergetics in penile arteries from ER-stress rats.

Several stresses including ischemia and oxidative stress lead to the disruption of ER homeostasis resulting in the accumulation of unfolded proteins and ER stress that triggers the UPR, activated in obesity and insulin resistance when the elevated protein synthesis demand due to the excess of energy substrates exceeds protein folding and post-translational maturation [1,21,22]. The current data show ER stress in penile erectile tissue of tunicamycin-treated rats, as revealed by the increased protein expression of the chaperone BiP and the main arms of the UPR (Figure 1). Thus, tunicamycin, a potent inhibitor of the first step of glycoprotein synthesis in the ER, induced up-regulation of phosphorylated branch of UPR PERK (Figure 1b), that attenuates mRNA translation to reduce the ER protein load, and increases expression of ATF6α that in turn induces expression of chaperones to assist in the correct protein folding and proteolytic cleavage. When the stress persists, hyperactivation of UPR increases ROS and oxidative stress, triggering inflammatory and apoptotic pathways involving IRE1α that lead to endothelial dysfunction [21,22]. Accordingly, tunicamycin treatment enhanced expression of BiP (Figure 1a), pPERK (Figure 1b), ATF6α (Figure 1c) and the phosphorylated form of IRE1α (Figure 1d) in rat erectile tissue.

ER stress disrupts Ca^2+^ homeostasis, a key regulator of vascular tone. Treatment with the ER-stress inductor tunicamycin blunted adrenergic vasoconstriction in penile arteries, consistent with previous findings in arteries from gluteal biopsies of patients with cerebral arteriopathy where the expression of ER-stress markers was associated with decreased Phe vasoconstriction [23], and also in rat liver vessels where ex vivo acute treatment with tunicamycin reduced vascular intracellular Ca^2+^ levels [24]. In penile arteries, ER stress significantly reduced contractions to the agonist of α_1_-adrenergic receptor Phe, coupled to phospholipase C (PLC)-IP_3_-induced sarcoplasmic reticulum (SR) intracellular Ca^2+^ release pathway, without altering vasoconstriction evoked by high K^+^-induced depolarization dependent on extracellular Ca^2+^ entry through voltage-dependent L-type channels [25]. Sarco/endoplasmic reticulum Ca^2+^ ATPase (SERCA) is the main regulator of ER Ca^2+^ homeostasis, and a reduction in SERCA activity has been reported to induce hepatic ER stress while SERCA overexpression in vivo attenuated chronic ER stress [26]. Therefore, blunted α_1-_adrenergic vasoconstriction induced by ER stress in penile arterial smooth muscle might be ascribed to impairment of the SR intracellular Ca^2+^ store.

ER stress is a key factor linking metabolic disease and vascular endothelial dysfunction, the initial step of atherosclerosis and cardiovascular disease. In the present study, in vivo ER-stress induction with tunicamycin was associated with endothelial dysfunction shown by the reduced NO-mediated relaxations elicited by the endothelial agonist ACh in penile arteries, as well as by the decreased relaxations to the β-adrenergic agonist isoprenaline that elicits vasodilation in part through phosphorylation and activation of eNOS via the endothelial PI3K/Akt pathway [27], the latter being blunted in penile arteries from genetically obese rats [12]. Both chemically-induced ER stress with tunicamycin and hypertension have been reported to impair endothelial vasodilatation and decreased eNOS phosphorylation and NO bioavailability in aorta and mesenteric resistance arteries, while ER-stress inhibition restored eNOS phosphorylation and improved NO-mediated endothelial relaxations in hypertensive mice [28]. In line with the present findings, diabetes mellitus leads to erectile dysfunction associated with ER stress, penile endothelial dysfunction, and enhanced expression of ER-stress markers including apoptotic proteins [29,30]. Since activation of the UPR in endothelial cells was reported to be also associated with blunted endothelial flow-mediated vasodilatation in obese non-diabetic individuals [31] and decreased eNOS phosphorylation and endothelial NO production in HFD-induced obese mice, penile endothelial dysfunction in tunicamycin-treated rats suggests that ER stress may contribute to penile endothelial dysfunction recently reported in obesity [18].

Vascular oxidative stress has been shown to be a downstream mechanism of ER stress [8,28] although oxidative stress can also induce ER stress, suggesting that oxidative stress and ER stress can activate each other [28,32,33]. In hypertension, diabetes, or tunicamycin-induced in vivo ER stress models, increased NADPH oxidase activity is a major source of oxidative stress associated with impaired endothelium-dependent relaxations in aorta and resistance arteries [8,28]. Enhanced gene expression of Nox2 and Nox4 and ROS production were reported to downregulate eNOS and NO levels via p38 MAPK activation in tunicamycin-challenged endothelial cells [8]. In the present study, selective inhibition of Nox1 improved NO-mediated endothelium-dependent relaxations in penile arteries of tunicamycin-treated rats suggesting that Nox1-derived superoxide contributes to endothelial dysfunction as earlier reported for renal arteries from both HFD-induced and genetically obese rats [17,34]. Interestingly, the use of the selective Nox2 antagonist Nox2ds-tat revealed Nox2-mediated endothelial relaxations in penile arteries of control rats, similarly to that earlier found in healthy human renal arteries [34]. ER stress blunted this Nox2-dependent endothelial relaxation in arteries from tunicamycin-treated rats, thus suggesting that loss of Nox2-derived vasodilator H_2_O_2_ also contributes to endothelial dysfunction, as reported in renal preglomerular arteries from genetically obese rats [34].

We have recently demonstrated that mitochondrial-derived ROS are involved in the endothelium-dependent relaxations of penile arteries under physiological conditions [18]. Since altered ER–mitochondria interactions may contribute to mitochondrial oxidative stress and subsequent mitochondrial dysfunction in metabolic diseases [3], the role of mtROS in penile endothelial dysfunction of tunicamycin-treated rats was further investigated. Here we demonstrate that ER stress blunts H_2_O_2_-mediated vasodilatation of penile arteries thus contributing to endothelial dysfunction, as depicted by the lack of inhibitory effect of the mitochondrial antioxidant mitoTEMPO on both ACh- and isoprenaline-induced relaxations. The loss of vasodilator mitochondrial H_2_O_2_ was further supported by the reduced mtROS and H_2_O_2_ levels found in both penile arteries and corpus cavernosum from ER-stress rats, along with a downward trend for expression of the transcriptional coactivator PGC-1α that regulates mitochondrial biogenesis and mitochondrial antioxidants systems, thus supporting a reduction of H_2_O_2_ levels induced by ER stress in erectile tissue. These findings support data recently reported in kidney preglomerular arteries wherein ER stress blunts mitochondrial H_2_O_2_ vasodilator levels and endothelial relaxations [19]. While impaired ER–mitochondria interactions in metabolic disorders have been involved in altered insulin signaling leading to insulin resistance [35], or impaired calcium signaling activating apoptotic pathways [3,36], here we demonstrate that abnormal mitochondria-ER interactions in the UPR lead to mitochondrial redox imbalance and reduced mitochondrial vasodilator mtROS, thus contributing to endothelial dysfunction in penile erectile tissue.

Although the mechanisms by which UPR triggers apoptotic pathways are well established, the metabolic events and the impact of UPR on mitochondrial bioenergetics during the adaptive phase of ER stress are less understood. In the present study, increased basal mitochondrial respiration, oxygen consumption for ATP synthesis, and glycolytic activity shown by the increased extracellular acidification rates and elevated blood lactate levels, were found in penile arteries from ER-stress rats associated with reduced mtROS levels and enhanced pIRE1α expression in erectile tissue, the latter suggesting that IRE1α activity may be related to mitochondrial bioenergetic output as earlier reported [37,38]. Activation of mitochondrial bioenergetics has been shown to be an adaptive response to ER stress that enhances oxygen consumption upon the increased ATP demand for the higher chaperone activity [39]; augmented IRE1α activity and calcineurin have been reported to contribute to this compensatory response against augmented mtROS and excess calcium entering the mitochondria from ER, to increase efficiency of ETC, thus reducing ROS accumulation and promoting cell survival [37]. Loss of IRE1α impairs adaptive respiratory response and promotes accumulation of mitochondrial ROS and cell death [37]. The present findings demonstrate reduced mtROS levels along with augmented mitochondrial respiration in penile erectile tissue from tunicamycin-treated rats, in agreement with that shown in-kidney preglomerular arteries from the same in vivo model of chemically-induced ER stress [17], and suggesting a metabolic adaptation in response to ER stress to reduce ROS production. On the other hand, increased proton leak along with enhanced mitochondrial respiration, as found in penile arteries from tunicamycin-treated rats, maybe ascribed to mechanisms reported to promote proton leak to reduce mtROS in a protective feed-back loop that limits damage to the mitochondria [40].

Mitochondrial respiration measurements in intact penile arteries indeed reflect whole artery metabolism, although the contribution of endothelial and VSM cells is probably different. Amount of endothelial cells in the arterial wall is lesser than that of VSM cells, and whereas endothelial cells mainly rely on anaerobic glycolysis and are involved in calcium homeostasis and cell signaling via generation of vasodilator ROS as shown in the present study, VSM cell mitochondria are involved in energy production via OXPHOS to mediate regulation of arterial tone, secretion, and transport [41]. Thus, arterial smooth muscle cells probably mainly account for the increase in oxygen consumed rates measured in intact penile arteries.

In summary, this study demonstrates that ER stress blunts adrenergic vasoconstriction, enhances Nox1-derived ROS and reduces mitochondrial vasodilator H_2_O_2_ both underlying penile endothelial dysfunction, and augments mitochondrial respiration in penile erectile tissue (Figure 6). Although further studies will elucidate the specific molecular mechanisms underlying ER-stress-induced penile vascular dysfunction, our data provide robust pharmacological and functional evidence—including accurate assessment of endothelial function, mtROS generation, and mitochondrial bioenergetics—in freshly isolated, intact penile microarteries under conditions of in vivo ER stress.

## 4. Materials and Methods

### 4.1. Animal Model

For ER-stress investigations, a total of 36 male Wistar rats (18 controls and 18 tunicamycin-treated) were used at the age of 16 weeks in 3 different sets of experiments, and randomly assigned in two groups of 5–7 rats/set that were treated with 0.35 mg/kg tunicamycin solution intraperitoneally every two days during two weeks or vehicle (ethanol 96% diluted 1:5 in saline) [17]. Animals were maintained in groups of three to four per cage under standard conditions (12:12-h light-dark cycle, 22 °C, 60% relative humidity). Rats were weighed every two days, and blood glucose, total triglycerides, and lactate levels were determined in blood obtained from rat tail by using specific tests with an Accutrend^®^ Plus meter (Roche Diagnostics, Mannheim, Germany). After two weeks of treatment, rats showed an increase in lactate levels compared to control rats (Table 1). ER-stress markers were determined in erectile tissue of control and tunicamycin-treated by Western blotting analysis.

Rats were euthanized by cervical dislocation followed by exsanguination. The penis and corpora cavernosa (CC) were excised and immediately immersed in cold (4 °C) physiological saline solution (PSS) composed of (in mM): 119 NaCl, 4.7 KCl, 1.18 KH_2_PO_4_, 1.17 MgSO_4_, 1.5 CaCl_2_, 24.9 NaHCO_3_, 0.027 EDTA, and 11 glucose, adjusted to pH 7.4. All procedures involving animals were conducted according to the European Union Directive 2010/63/EU for the protection of animals used for scientific purposes. The procedures were also approved by the Institutional Animal Care and Use Committee of Comunidad de Madrid (Ref. PROEX 192.3/20; PROEX 230.1/23).

Dorsal penile arteries were carefully isolated by removing surrounding connective tissue and mounted in parallel in four-channel microvascular myographs (Danish Myotechnology, Hinnerup, Denmark) for isometric tension recording. Vessels were equilibrated for 30 min at 37 °C in PSS, continuously bubbled with a gas mixture of 95% O_2_ and 5% CO_2_ to maintain pH stability. Subsequently, the internal circumference L100 equivalent to a transmural pressure of 100 mmHg in a relaxed artery, and L_1_ = 0.9 × L100, at which maximal force generation occurs, were calculated. From L1, the internal diameter (l_1_) was determined individually for each vessel.

Additionally, small sections of both dorsal penile arteries and CC tissue were collected for mitochondrial reactive oxygen species (mtROS) analysis and western blot experiments. Freshly isolated segments of penile arteries were also used for mitochondrial respiration assessments.

### 4.2. Experimental Procedure for the Functional Experiments

At the beginning of each experiment, vasoconstrictor responses were assessed by challenging arteries with a high-potassium physiological saline solution (KPSS), formulated by replacing NaCl with equimolar KCl to achieve a final K^+^ concentration of 123.7 mM. Endothelial function was evaluated by measuring relaxation responses to acetylcholine (ACh; 0.01–30 µM) in vessels precontracted with phenylephrine (Phe) (0.3–3 μM). Vasoconstrictor responsiveness was further assessed through concentration–response curves (CRCs) to Phe (0.01–30 µM).

To investigate the role of mitochondrial reactive oxygen species (mtROS) in endothelium-dependent vasodilation of penile arteries from tunicamycin-treated rats, CRCs to ACh (0.01–30 µM) were generated in the absence and presence of the mtROS scavenger mitoTEMPO (0.3 µM) (ThermoFisher Scientific, Madrid, Spain) [17,18] or the non-selective NADPH oxidase (Nox) inhibitor apocynin (10 µM) in arteries from the experimental rat group (ER stress) and compared to controls. The effects of the selective Nox1 inhibitor NoxA1ds (0.1μM) (MedChemExpress, Monmouth Junction, NJ, USA) [34], the dual Nox1/4 inhibitor GKT136901 (0.1 μM) (MedChemExpress, Monmouth Junction, NJ, USA) [34], and the selective Nox2 inhibitor Nox2ds-tat (1 μM) (MedChemExpress, Monmouth Junction, NJ, USA) [34], were also evaluated on the endothelial relaxations of penile arteries from tunicamycin-treated rats. Following the initial CRC, arteries were incubated for 30 min with either mitoTEMPO or a Nox inhibitor, after which a second CRC was conducted in the continuous presence of the respective inhibitor in the myograph chamber. PE-induced contraction in the second CRC was matched to that in the control curve.

### 4.3. mtROS Measurements

mtROS production was assessed using the mitochondria-specific hydroethidine derivative, MitoSOX [42]. Freshly isolated segments of penile arteries and corpora cavernosa (CC) were equilibrated in HEPES-buffered physiological saline solution (PSS) for 30 min at 37 °C. Tissues were then incubated in black-walled microtiter plates containing 15 µM MitoSOX (Molecular Probes, ThermoFisher Scientific, Madrid, Spain) for 30 min at 37 °C. Fluorescence intensity was recorded using a fluorometer (BMG Fluostar Optima, Ortenberg, Germany) with an excitation wavelength of 510 nm and an emission wavelength of 580 nm. Background fluorescence was subtracted, and mtROS levels were normalized to the dry weight of the tissue samples.

Hydrogen peroxide (H_2_O_2_) concentrations were determined using the Amplex Red assay kit (Life Technologies, ThermoFisher Scientific, Madrid, Spain) in penile arteries and CC isolated from ER-stress and control animals, following established protocols for intact small vessels [34]. Samples were equilibrated in HEPES-PSS for 30 min at room temperature, and subsequently transferred to black-walled microtiter plates containing Amplex Red at a final concentration of 100 µM and horseradish peroxidase at 10 U/mL in reaction Buffer. Fluorescence measurements were performed using a BMG Fluostar Optima fluorometer, with excitation at 544 nm and emission at 590 nm. Background fluorescence was subtracted, and H_2_O_2_ production was normalized to tissue dry weight.

### 4.4. Western Blotting Analysis

CC tissues from tunicamycin-treated (ER stress), and their corresponding vehicle-treated control groups were rapidly snap-frozen in liquid nitrogen, homogenized, and lysed in a buffer containing 50 mM Tris-HCl (pH 7.5), 1 mM EGTA, 1mM EDTA, 1% (*v*/*v*) Triton X-100, 0.1 mM sodium orthovanadate, 50 mM sodium fluoride, 5 mM sodium pyrophosphate, 0.27 M sucrose plus a protease inhibitor cocktail. Protein concentrations were quantified using the Bradford assay (Bio-Rad Laboratories, S.A., Madrid, Spain). It is worth highlighting that a very limited amount of protein can be obtained from rat penile CC samples. A total of 5–7 µg of protein lysate was resolved on a 6.5% SDS-polyacrylamide gel, transferred onto a polyvinylidene difluoride (PVDF) membrane, and subsequently incubated with the following primary antibodies against mitochondrial redox metabolism enzymes manganese superoxide dismutase (MnSOD) and against the peroxisome proliferator-activated receptor γ coactivator 1α (PGC-1α) (Abcam, Cambridge, UK), ER-stress markers ATF6α, pPERK (Thr981), peIF2α (Ser52), pIRE1α (Ser724) (Abcam, Cambridge, UK), and GRP78 (BiP) (Cell Signaling, Danvers, MA, USA); and β-actin (Sigma, St. Louis, MO, USA). Bound primary antibodies were detected with horseradish peroxidase conjugated to secondary antibodies. Blots were visualized by enhanced chemiluminescence and quantified by densitometry with the free ImageJ software, 1.51m9 version. Levels of protein expression were normalized to β-actin and expressed as percentage of the control group mean. 

### 4.5. Mitochondrial Respiration Measurements

Mitochondrial oxygen-consumption rate (OCR) and extracellular-acidification rate (ECAR) were measured using the Agilent Seahorse XF HS Analyzer to evaluate mitochondrial respiration, oxidative phosphorylation (OXPHOS), and glycolytic activity in real-time with the MitoStress Assay. Freshly isolated penile dorsal arteries from control and tunicamycin-treated rats were used for these experiments, as previously described [18]. Briefly, arterial segments approximately 2 mm in length were placed at the bottom of wells in an XF HS Islet Capture Microplate. Each well was filled with 250 µL of XF assay medium supplemented with 5.0 mM glucose, 2 mM pyruvate, and 2 mM glutamine, and incubated at 37 °C in a non-CO_2_ incubator for 20 min prior to measurements.

Oxygen and proton concentrations in the surrounding medium were repeatedly recorded using the oxygen- and hydrogen ion-selective fluorophores integrated into the Seahorse XF HS Analyzer, enabling the calculation of OCR (pmol O_2_ min^−1^) and ECAR (millipH min^−1^). The *MitoStress* assay protocol involved four baseline measurement cycles, followed by four cycles after each sequential treatment. Wells were exposed to final concentrations of 50 µM oligomycin (to inhibit ATP synthase and block OXPHOS), 50 µM FCCP (carbonyl cyanide 4-(trifluoromethoxy) phenylhydrazone; a protonophore that induces maximal mitochondrial respiration by collapsing the membrane potential), and 10 µM rotenone/antimycin A (complex I and III inhibitors, respectively, impairing mitochondrial electron transport and membrane potential).

Due to the use of intact penile-artery segments rather than isolated cells, higher concentrations of oligomycin, FCCP, and rotenone/antimycin A were required to ensure effective penetration across the arterial wall. Throughout the experiment, chamber oxygen levels were continuously monitored to prevent hypoxic conditions. OCR and ECAR values were normalized to the surface area of the artery segments (expressed as mm^2^).

### 4.6. Data Presentation and Statistical Analysis

In myograph studies, arterial mechanical responses were recorded as force and subsequently expressed as active wall tension (ΔT), calculated by dividing the change in force (ΔF) by twice the length of the vessel segment. Tension values were presented either in newtons per meter (N·m^−1^) or as a percentage relative to the maximal contraction induced by KPSS or a specific vasoconstrictor in each artery. Vascular sensitivity to contractile and relaxant agents was assessed by determining pD_2_ values, defined as the negative logarithm of the agonist concentration causing 50% of the maximal response (EC_50_). EC_50_ values were obtained by fitting individual CRCs to the Hill equation through non-linear regression analysis using Prism 8.0 software (GraphPad, San Diego, CA, USA). The mean EC_50_ value for each experimental group was calculated after obtaining individual values.

Data are presented as mean ± standard error of the mean (SEM); “*n*” denotes the number of arteries analyzed, each from a minimum of four different animals. Statistical comparisons between groups were performed using unpaired Student’s *t*-test, depending on the experimental design. A *p*-value < 0.05 was considered statistically significant. Significant differences between more than two experimental conditions were analyzed using one-way ANOVA followed by Tukey’s multiple comparisons test. All analyses were conducted using Prism 8.0 (GraphPad Software, San Diego, CA, USA).

## 5. Conclusions

The present results demonstrate that Nox-1-derived oxidative stress along with impaired ER–mitochondria interactions leading to mitochondrial-redox imbalance and blunted mitochondrial H_2_O_2_ levels contribute to endothelial dysfunction in penile erectile tissue. Increase in mitochondrial respiration and glycolytic activity associated with enhanced IRE1α activity may downregulate mitochondrial ROS production and oxidative stress, thus ameliorating NO-mediated endothelial dysfunction. Inhibition of ER stress with chemical chaperones and endogenous bile acids that regulate ER function have been shown to improve cardiac fibrosis and endothelium-dependent vasodilation in hypertension [20,28]. Thus, attenuation of ER stress emerges as a potential novel therapeutic target in the management of metabolic disease-associated endothelial and erectile dysfunction.

## Figures and Tables

**Figure 1:**
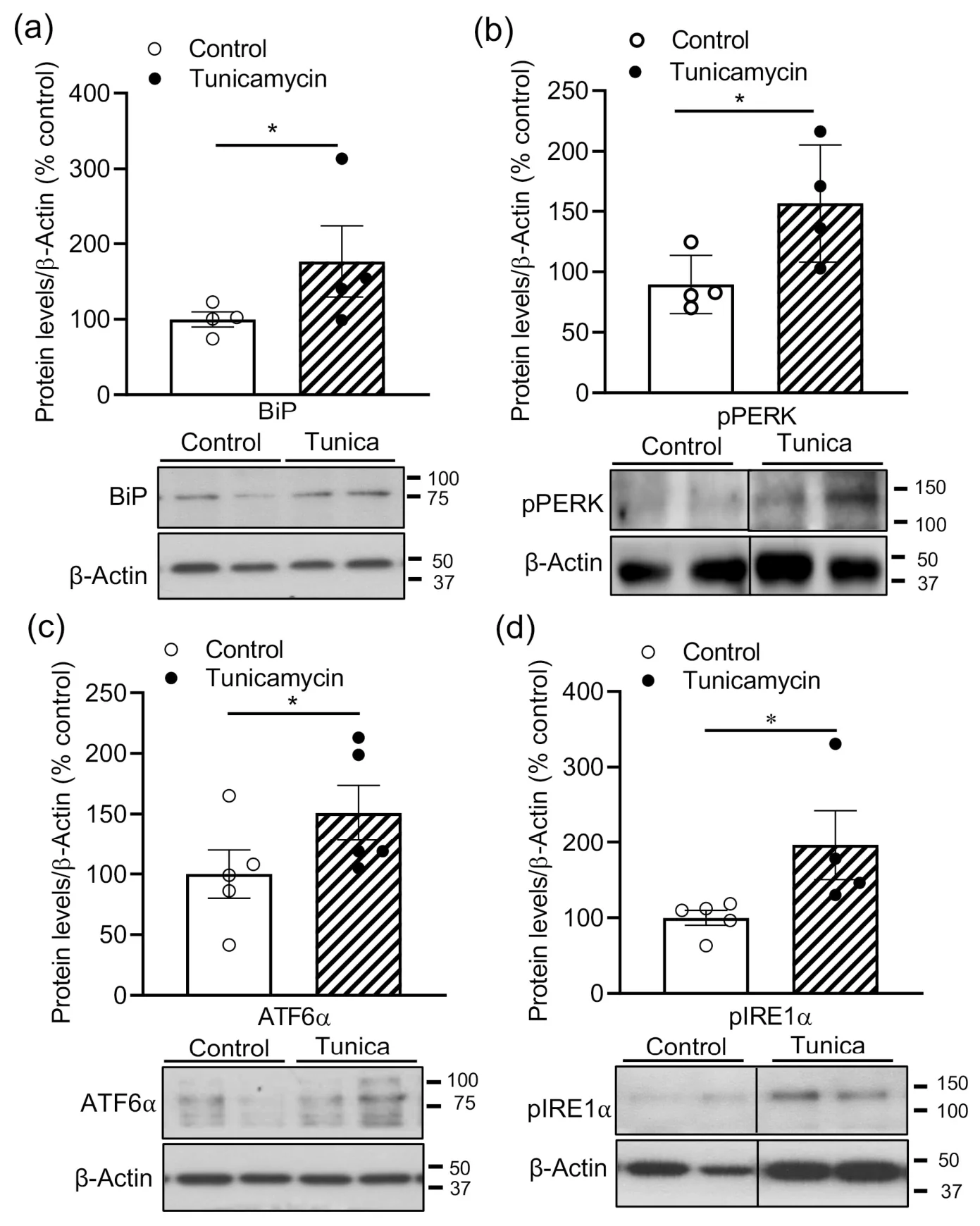
ER-stress markers in the corpus cavernosum (CC) of tunicamycin-induced ER-stress rats. Western blot analysis of BiP (**a**), pPERK (**b**), ATF6α (**c**), and pIRE1α (**d**) expression in CC samples from control and tunicamycin-treated rats. Levels of BiP, pPERK, ATF6α, and pIRE1α were increased in CC samples from ER-stress rats compared to controls. Results were quantified by densitometry and presented as the ratio of the density of the BiP, pPERK, ATF6α, and pIRE1α bands to those of β-actin in the sample. Results are expressed as the mean ± SEM of CC samples from *n* = 4–5 control and 4–5 tunicamycin-treated rats. * *p* < 0.05 significantly different from control arteries, Student’s *t*-test for unpaired observations.

**Figure 2:**
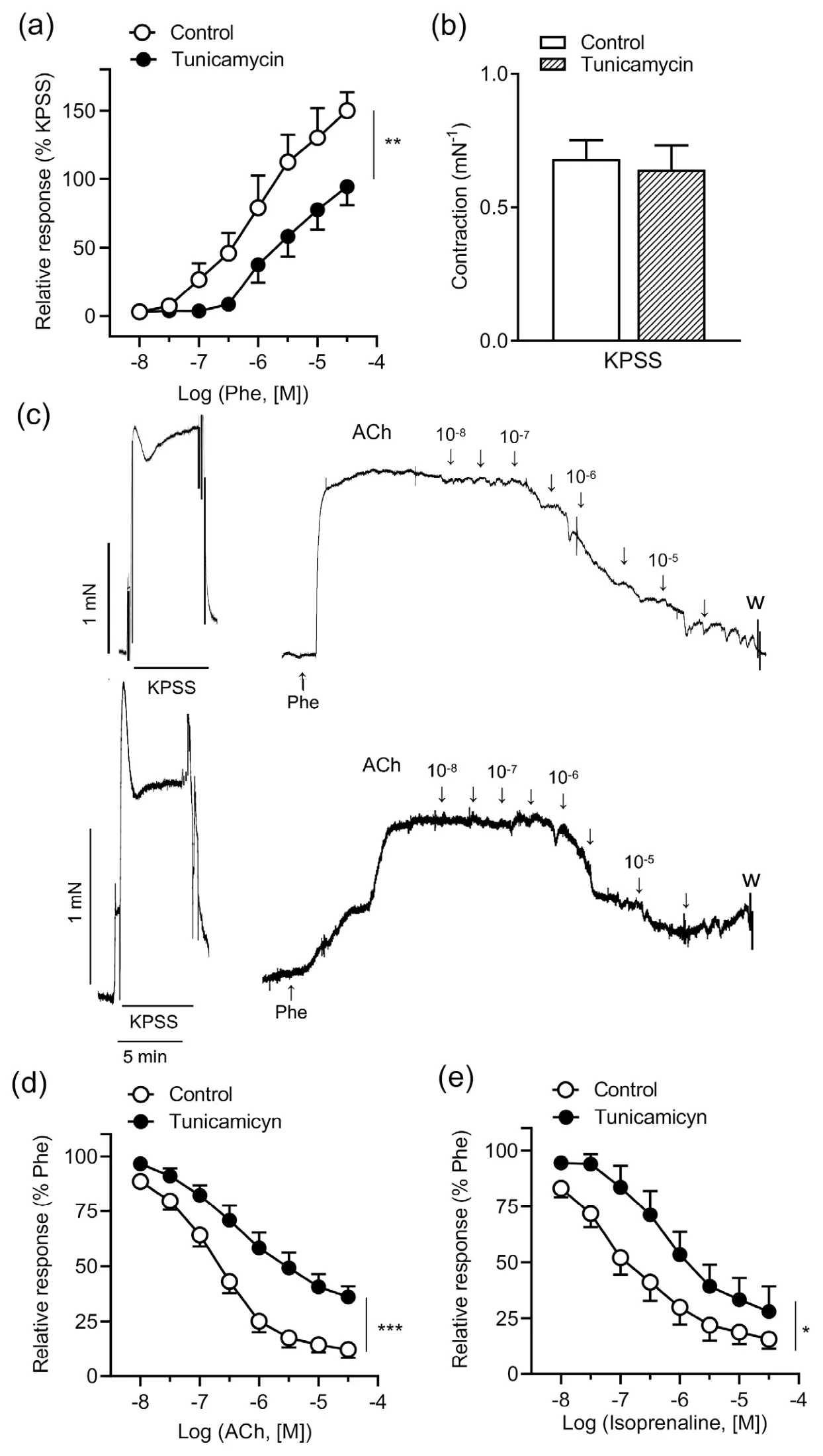
Chemically-induced ER stress blunts α_1_-adrenergic vasoconstriction and induces endothelial dysfunction of penile arteries. (**a**,**b**) Average contractions induced by the α_1_-adrenergic agonist phenylephrine (Phe) (**a**) and by high K^+^ depolarization (**b**) in control and tunicamycin-treated penile arteries. The results are expressed as a percentage of the KPSS contraction (**a**) and tension (mN mm^−1^) (**b**). Data are shown as the mean ± SEM of seven control arteries and eleven tunicamycin-treated arteries (1–2 arteries/animal). ** *p* < 0.01 significantly different from control arteries, Student’s *t*-test for unpaired observations. (**c**–**e**) Effect of ER stress on the vasodilatory responses of penile arteries. (**c**) Isometric force recordings showing the relaxations to the endothelial agonist ACh in penile arteries from a control rat (**c**, top panel) and from a tunicamycin-treated rat (**c**, bottom panel) w, washing. (**d**,**e**) Average relaxations of control and ER-stress penile arteries to ACh (**d**) and isoprenaline (**e**). Tunicamycin induced a significant impairment in vasodilatory responses. Data are presented as mean ± SEM of 15 arteries (1–2 arteries/animal) (**d**) and 6–8 arteries (1–2 arteries/animal) (**e**). * *p* < 0.05, ** *p* < 0.01; *** *p* < 0.001 vs. control, unpaired Student’s *t*-test.

**Figure 3:**
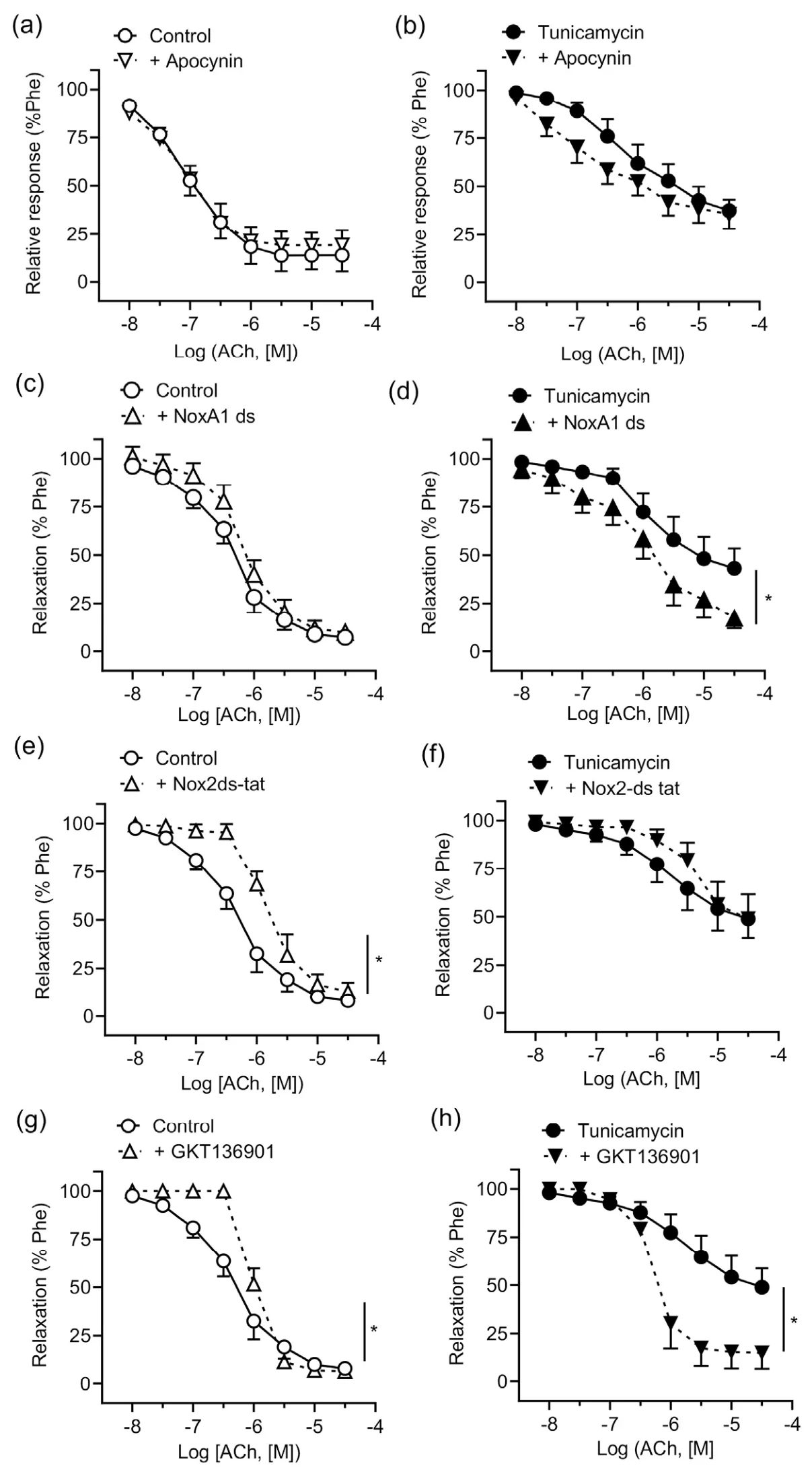
Effect of Nox inhibitors on penile endothelial dysfunction induced by ER stress. Average relaxations of control (**a**,**c**,**e**,**g**) and tunicamycin-treated (**b**,**d**,**f**,**h**) penile arteries to the endothelial agonist ACh, and effects of the non-selective Nox inhibitor apocynin (**a**,**b**), the selective Nox1 inhibitor Nox1A ds (**c**,**d**). the selective Nox2 inhibitor Nox2 ds-tat (**e**,**f**) and the Nox1/Nox4 inhibitorGKT136901 (**g**,**h**). Data are presented as mean ± SEM of *n* number of arteries from 5–6 animals (1–2 per animal). Statistical differences were calculated by using one-way ANOVA followed by Tukey’s multiple comparisons test. * *p* < 0.05 vs. control.

**Figure 4:**
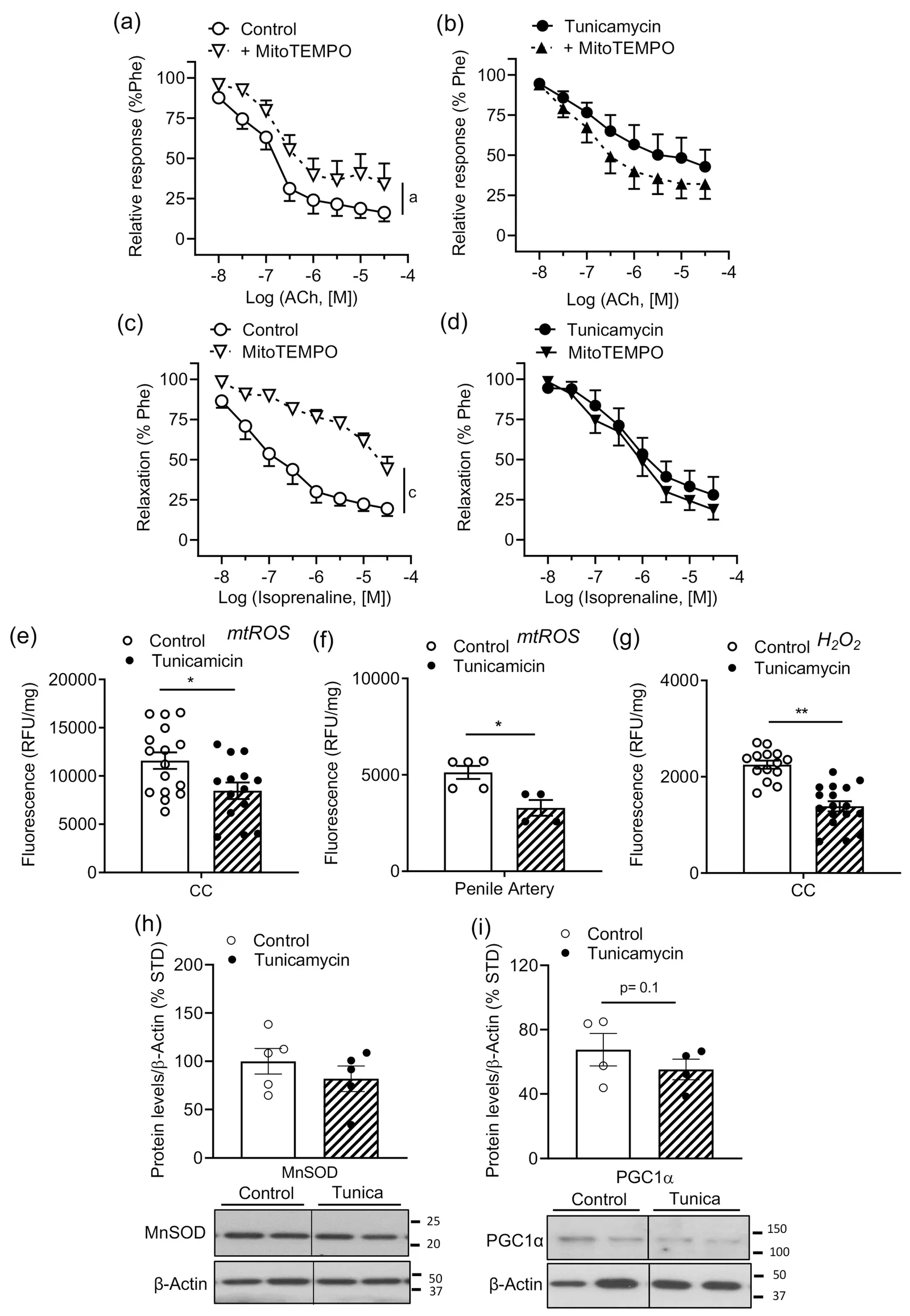
Effect of the mitochondrial antioxidant mitoTEMPO on penile endothelial dysfunction and mtROS levels in erectile tisssue from ER-stress rats. Average relaxations of control (**a**,**c**) and tunicamycin-treated (**b**,**d**) penile arteries to the endothelial agonist ACh (**a**,**b**) and to the β-adrenergic agonist isoprenaline (**c**,**d**), and effects of mitoTEMPO on vehicle- (**a**–**c**) and tunicamycin-treated (**b**,**d**) arteries. Data are presented as mean ± SEM of six (**a**,**b**), and seven (**c**,**d**) arteries (1–2 per animal). Statistical differences were calculated using one-way ANOVA followed by Tukey’s multiple comparisons test. ^a^ *p* < 0.05 vs. control. ^c^ *p* < 0.001 vs. control. (**e**–**g**) Mitochondrial superoxide levels measured with mitoSOX in CC (**e**) and penile artery (**f**) samples from control and tunicamycin-treated rats. (**g**) Basal H_2_O_2_ levels measured with Amplex Red in CC from control and ER-stress rats. Results are expressed as relative fluorescence units (RFU) per mg of tissue. (**h**,**i**) Western blot analysis of MnSOD and PGC1α expression in CC samples from control and tunicamycin-treated rats. Results were quantified by densitometry and presented as the ratio MnSOD and PGC1α band densities to β-actin in the sample. Results are expressed as mean ± SEM of 16 control CC samples and 14 tunicamycin-treated CC samples from *n* = 8 rats (**e**), *n* = 5 rats for both control and tunicamycin-treated arteries (**f**), 15 control CC samples, and 18 tunicamycin samples from *n* = 9 rats (**g**), (**h**,**i**) For Western blot analysis, CC samples from *n* = 4–5 control rats and *n* = 4–5 tunicamycin rats. * *p* < 0.05, ** *p* < 0.01 significantly different from control samples, Student’s *t*-test for unpaired observations.

**Figure 5:**
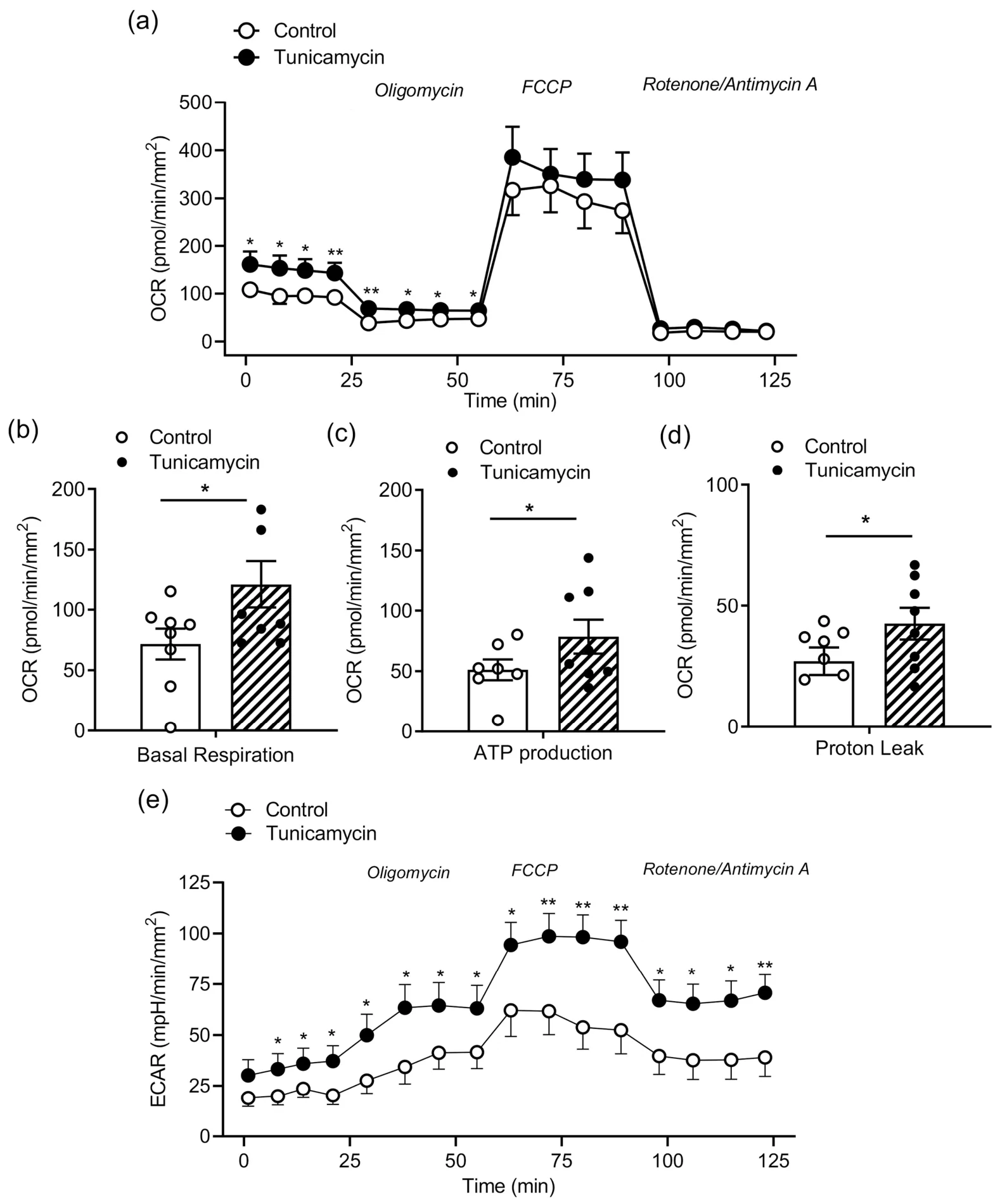
Mitochondrial respiratory profile of penile arteries from rats with ER stress. Oxygen consumption rate (OCR) of isolated penile arteries from control rats and ER-stress rats (**a**). Mitochondrial bioenergetic parameters are shown as basal respiration (**b**), oxygen consumed for ATP production (**c**), proton leak (**d**), and ECAR (**e**), an indicator of glycolytic activity. Results are expressed as values in pmol O_2_· min^−1^, normalized to the arterial segment surface area in mm^2^. Data are presented as mean ± SEM of arteries from *n* = 7 control and *n* = 7–8 tunicamycin-treated rats. * *p* < 0.05, ** *p* < 0.01, significantly different from control arteries, Student’s *t*-test for unpaired observations.

**Figure 6:**
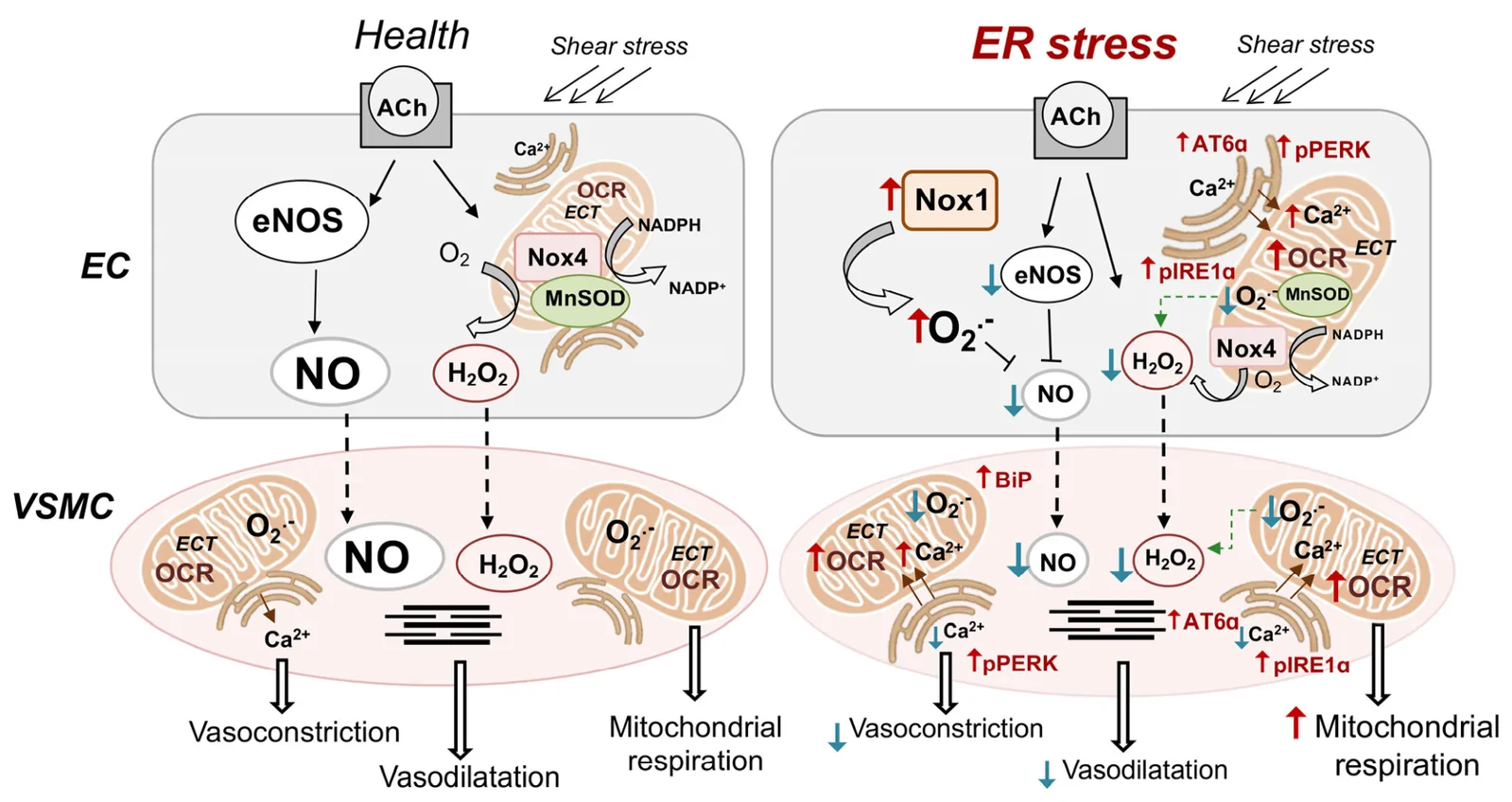
ER stress-induced penile endothelial dysfunction, impaired REDOX balance, and enhanced mitochondrial respiration. *See Discussion for details*. Compartmentalization of ROS under ER-stress–cytosolic Nox1-derived ROS, and mitochondrial-derived H_2_O_2_. ACh, acetylcholine; ATF6 activating transcription factor 6; BiP, binding immunoglobulin protein; EC, endothelial cell; pIRE1α phosphorylated endoribonuclease inositol-requiring enzyme 1α; ETC: electron-transport chain; H_2_O_2_: hydrogen peroxide; PKR, protein kinase R; pPERK phosphorylated PKR-like endoplasmic reticulum kinase; ER stress, endoplasmic-reticulum stress; NO, nitric oxide; eNOS: endothelial NO synthase; Nox1: NADPH oxidase 1; Nox4: NADPH oxidase 4; MnSOD: manganese superoxide dismutase; OCR: oxygen consume rate; VSMC, vascular smooth muscle cell. 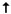 red arrows indicate increase; 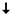 green arrows indicate decrease. 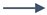 Measured 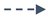 Inferred.

**Table 1:** Metabolic parameters of vehicle and tunicamycin-treated rats.

	Vehicle	*n*	Tunicamycin	*n*
Weight (g)	339 ± 21	6	315 ± 46	5
Age (weeks)	16	-	16	-
Glucose _Blood_ (mg/dL)	151 ± 7.0	19	187 ± 19 **^†^**	17
Lactate _Blood_ (mg/dL)	2.88 ± 0.19	17	3.99 ± 0.65 *	12
Triglycerides _Blood_ (mg/dL)	135 ± 10	18	139 ± 8.4	15

Values of weight, age, and biochemical parameters in control rats and rats with ER stress induced by tunicamycin. The data are expressed as means ± SEM for the weight of six control rats and five ER-stress rats, as well as 1–2 replicates of blood samples measurements from nine control and nine ER-stress rats, respectively, for glucose, lactate, and triglycerides. * *p* < 0.05 significantly different from control arteries, Student’s *t*-test for unpaired observations. ^†^ *p* = 0.07.

**Table 2:** Effects of Nox inhibitors on the endothelium-dependent relaxations to ACh of penile arteries from vehicle and tunicamycin-treated rats.

	pD_2_	E_max_ (% Phe)	l_1_	*n*
Control	6.75 ± 0.11	82 ± 11	127 ±11	7
+ apocynin	6.68 ± 0.16	75 ± 10	127 ± 11	7
Tunica	5.61 ± 0.30 ^a^	63 ± 7	135 ± 10	9
+ apocynin	6.43 ± 0.37	64 ± 8	135 ± 10	9
Control	6.40 ± 0.15	92 ± 2	175 ± 9	10
+ NoxA1 ds	6.22 ± 0.11	90 ± 3	175 ± 9	10
Tunica	5.67 ± 0.23 ^b^	57 ± 10 ^b^	145 ± 15	7
+ NoxA1ds	5.99 ± 0.22	79 ± 6 ^c^	145 ± 15	7
Control	6.57 ± 0.10	91 ± 2	157± 10	7
+ Nox2ds-tat	5.93 ± 0.03 ^a^	87 ± 4	157 ± 10	7
Tunica	5.67 ± 0.23 ^b^	57 ± 10 ^b^	145 ± 15	7
+ Nox2ds-tat	5.32 ± 0.15	51 ± 13 ^b^	145 ± 15	7
Control	6.48 ± 0.14	96 ± 2	177 ± 16	5
+ GKT 136901	6.00 ± 0.05 ^a^	93 ± 2	177 ± 16	5
Tunica	5.92 ± 0.20 ^b^	46 ±10 ^b^	205 ± 9	5
+ GKT 136901	6.27 ± 0.03	85 ± 8 ^c^	205 ± 9	5

Data are expressed as means ± SEM; *n* is the number of arteries (1–2 per animal) from 5–6 animals. The results are expressed as relative values, as percentages of the contractile response to phenylephrine (Phe). + means the addition of a treatment to the group indicated on the line immediately above. pD_2_ is –log(EC_50_), where EC_50_ is the agonist concentration that produces 50% of the maximum response (E_max_). Significant differences were analyzed using Statistical differences and were calculated using one-way ANOVA followed by Tukey’s multiple comparisons test. ^a^ *p* < 0.05 vs. control ^b^ *p* < 0.001 vs. Control. ^c^ *p* < 0.05 vs. tunicamycin treated; Arteries were incubated and exposed only once to the corresponding Nox inhibitor before a CRC for ACh was repeated.

**Table 3:** Effects of the mitochondrial antioxidant mitoTEMPO on the relaxations to acetylcholine (ACh) and isoprenaline of penile arteries from vehicle and tunicamycin treated rats.

*Acetylcholine*				
	pD_2_	E_max_ (% Phe)	l_1_	*n*
Control	6.97 ± 0.16	83 ± 5	127± 11	8
+ mitoTEMPO	5.77 ± 0.43 ^a^	66 ± 13	127 ± 11	8
Tunica	5.91 ± 0.34	64 ± 12	136 ± 9	7
+ mitoTEMPO	6.62 ± 0.22	77 ± 8	136 ± 9	7
***Isoprenaline***				
	pD_2_	E_max_ (% Phe)	l_1_	*n*
Control	6.69 ± 0.26	80 ± 5	107 ±5	7
+ mitoTEMPO	4.86 ±0.16 ^b^	56 ± 8 ^a^	107 ± 5	7
Tunica	6.16 ± 0.18 ^c^	71± 11	135 ± 10	9
+ mitoTEMPO	6.27 ± 0.24	82 ± 6	135 ± 10	9

Data are expressed as means ± SEM; *n* is the number of arteries (1–2 per animal) from six animals. The results are expressed as relative values, as percentages of the contractile response to phenylephrine (Phe). + means the addition of a treatment to the group indicated on the line immediately above. pD_2_ is −log(EC_50_), where EC_50_ is the agonist concentration that produces 50% of the maximum response (E_max_). Significant differences were analyzed using Statistical differences were calculated using one-way ANOVA followed by a Tukey’s multiple comparisons test. ^a^ *p* < 0.05 vs. control ^b^ *p* < 0.001 vs. Control, ^c^ *p* < 0.05 vs. control.

## Data Availability

The data presented in this study are available upon reasonable request from the corresponding authors.

## Abbreviations

ATF6α	Activating transcription factor 6α
ATP	Adenosine triphosphate
CVD	Cardiovascular disease
ECAR	Extracellular acidification rate
eNOS	Endothelial nitric oxide synthase
ED	Erectile dysfunction
ER	Endoplasmic reticulum
ETC	Electron transport chain
MAPK	Mitogen-activated protein kinase
MCP-1	Monocyte chemoattractant protein-1
MnSOD	Manganese superoxide dismutase
mtROS	Mitochondrial reactive oxygen species
NO	Nitric oxide
NOS	Nitric oxide synthase
Nox	NADPH oxidase
OCR	Oxygen consumption rate
pIRE1α	Phosphorylated inositol-requiring enzyme 1α
PKR	Protein kinase R
pPERK	Phosphorylated PKR-like endoplasmic reticulum kinase
ROS	Reactive oxygen species
SERCA	Sarcoplasmic/Endoplasmic reticulum calcium ATPase
SR	Sarcoplasmic reticulum
UPR	Unfolded protein response
